# Increased Response to a 5-HT Challenge After Discontinuation of Chronic Serotonin Uptake Inhibition in the Adult and Adolescent Rat Brain

**DOI:** 10.1371/journal.pone.0099873

**Published:** 2014-06-17

**Authors:** Anne Klomp, Ralph Hamelink, Matthijs Feenstra, Damiaan Denys, Liesbeth Reneman

**Affiliations:** 1 Department of Radiology, Academic Medical Centre, University of Amsterdam, Amsterdam, the Netherlands; 2 Netherlands Institute for Neuroscience, Royal Netherlands Academy of Arts and Sciences, Amsterdam, the Netherlands; 3 Department of Psychiatry, Academic Medical Centre, University of Amsterdam, Amsterdam, the Netherlands; Nathan Kline Institute for Psychiatric Research and New York School of Medicine, United States of America

## Abstract

Little is known about the effects of chronic fluoxetine on 5-HT transmission in the adolescent brain, even though it is acknowledged that the neuroplasticity of the brain during childhood and adolescence might influence the neurobiological mechanisms underlying treatment response. Also, possible ongoing effects on monoamine function following drug discontinuation are unidentified. We therefore examined the chronic effects of fluoxetine on extracellular 5-HT and dopamine concentrations in the medial prefrontal cortex and studied their responsiveness to an acute 5-HT challenge after a one-week washout period, both in adolescent and adult rats. Noradrenaline was measured in adult animals only. Fluoxetine increased 5-HT to 200–300% of control and DA and NA to 150% of control. Although there were no lasting effects of chronic fluoxetine on basal monoamine levels, we observed a clear potentiating effect of previous treatment on the fluoxetine-induced increase in extracellular 5-HT and, to a lesser extent, extracellular DA. No differential effect was found for noradrenaline. Age-at-treatment did not influence these results. So, after cessation of chronic fluoxetine treatment 5-HT responsiveness remains heightened. This may be indicative of the continuing presence of 5-HT receptor desensitization, at least until one week after drug discontinuation in rats. No apparent age-at-treatment effects on extracellular monoamine concentrations in the medial prefrontal cortex were detected, but age-related differences in 5-HT transmission further down-stream or in the recovery processes cannot be ruled out.

## Introduction

The first onset of mood- and anxiety disorders usually occurs during (early) adolescence. It has therefore been suggested that early treatment of these disorders needs to focus more on youth. At the same time, in order to unravel its underlying mechanisms, research should lay more emphasis on the developing brain instead of the adult brain [1], [2]. Indeed, prescription rates of psychotropic drugs such as antidepressants have increased significantly in the last 20 years among children and adolescents [3], indicating better recognition of childhood depression. On the other hand, this expansion is worrisome since the ongoing neuroplasticity of the brain during childhood and adolescence might influence the neurobiological mechanisms underlying treatment response [4]. Indeed, the efficacy of selective serotonin (5-HT) reuptake inhibitors (SSRIs) in the treatment of pediatric depression is still disputed [5]–[9] while tricyclic antidepressants have been shown to be ineffective [10]. In addition, clinical studies have raised concerns about increases in suicidal ideation and behavior in children and adolescents treated with SSRIs [11]–[13] and also increased agitation, depression and anxiety [12] as well as negative effects on growth rate [14] have been described. This apparent heightened sensitivity to adverse effects has even led to governmental warnings on the use of antidepressants in youngsters. Nevertheless, the SSRI fluoxetine (FLX) was recently approved by the same governmental institutions for the treatment of childhood depression in children of 8 years and older.

Preclinical studies investigating the effects of antidepressant drugs on the developing brain still are in short supply, although several lines of research suggest that SSRIs have the potency to alter neurotransmitter development through their effect on 5-HT transmission and to affect consequently also neuronal outgrowth and maturation [4], [15]. Understandably, these effects will be most profound during early (perinatal) development, but the brain also undergoes extensive remodeling from youth through adolescence into adulthood, with 5-HT playing a central role in these processes [16], [17]. Although there are some preclinical studies that have investigated the long-term effects of antidepressant use in juvenile animal models, focus lies primarily on behavior, instead of on the more direct effects on neurotransmitter function [4], [17]. A recent pharmacological magnetic resonance imaging (phMRI) study by our own group clearly indicated altered 5-HT function following chronic FLX treatment, which was dependent on the age at treatment [18]. While adult-treated animals showed a decreased response in brain activity to an acute 5-HT challenge, this response was heightened in the adolescent-treated animals. The underlying pathways of these findings currently remain unclear. However, age-related effects of SSRI treatment on 5-HT transporter (SERT) density [19], [20], on markers of both 5-HT and dopamine (DA) function [21], and on dendritic spine proliferation [22] have been described before, all indicative of altered 5-HT function after chronic treatment in juveniles.

Extracellular 5-HT concentrations reflect changes in 5-HT activity and increases in these concentrations are known to modulate the MRI signal in the rat brain [23]. SSRIs enhance the extracellular concentrations of 5-HT by blocking the 5-HT transporters responsible for reuptake of 5-HT into the presynaptic terminal. Chronic administration results in higher levels of extracellular 5-HT than single administration [24]. Negative feedback loops that stabilize extracellular 5-HT concentrations are mainly regulated by the presynaptic 5-HT autoreceptors [25], [26], which can quickly attenuate the 5-HT increase caused by SSRIs [27], [28]. Chronic administration of SSRIs desensitizes these autoreceptors and leaves the 5-HT system more vulnerable to external influences [29]. Although the direct effects of chronic FLX on extracellular 5-HT concentrations have been extensively studied, little is known about its longer lasting effects. Moreover, it is unknown whether these effects are the same for the developing and developed brain. In view of the evidence mentioned above, such as the assumed age-dependent effects of chronic treatment on SERT density, it is not unlikely that the effects of chronic FLX on extracellular monoamine concentrations are also age-related.

The objective of this study was to determine if there are ongoing effects of chronic FLX after drug discontinuation on extracellular monoamine concentrations in adult- and adolescent-treated rats. Our aim was twofold; on one hand we wanted to gain more knowledge on the long-term effects of FLX on extracellular monoamine concentrations and on the other hand we liked to know if these long-lasting effects differ in relation to the age-of-treatment. To that end, adolescent and adult rats were chronically treated with FLX and, after a one-week washout period, extracellular 5-HT concentrations in the medial prefrontal cortex (mPFC) were measured before and after an acute FLX challenge using microdialysis. The acute challenge should significantly elevate the levels of 5-HT, DA, and noradrenaline (NA) in the mPFC [24], [30], [31]. Based upon earlier studies by Invernizzi (1996), we expected the elevated 5-HT concentrations directly after treatment to be normalized after a one-week washout period in the adult-treated animals, whereas the effects might be longer lasting or even permanent in adolescent-treated animals. Without any pretreatment, age differences in extracellular 5-HT concentrations between adolescent and adult rats were not expected [32].

## Materials and Methods

All experiments were carried out in accordance with the Dutch regulations governing animal welfare and protection. Protocols were reviewed and consented by the Committee on the Ethics of Animal Experiments of the Academic Medical Center of Amsterdam (registered under project number: DDR102588), in accordance with the guidelines of the European Communities Council Directive of 24 November 1986 (86/609/EEC). All invasive procedures were performed under anesthesia, and all efforts were made to minimize suffering and to limit the total amount of animals used.

### Animals

Male Wistar rats (N = 32) were obtained from Charles River Inc. (Germany). Age of arrival was either post-natal day 21 (PND21; ‘adolescent group’, 50–60 g) or PND61 (±3 days; ‘adult group’, 290–310 g). Within each age category, there was one experimental and one control group (n = 8). All rats were group-housed during treatment, until stereotactic surgery (4 animals per cage, type IV cage (1875 cm^2^)), under standard housing conditions and a 12 hour light-dark cycle. Food and water were available *ad libitum*. Water intake and bodyweight were monitored on a daily base.

### Drugs and Treatment

After an acclimatization period of four days, drug treatment was started at either PND25 or PND65 (±3 days). PND25 was selected because it approximates the start of adolescence in humans. In male rats, adolescence lasts from PND28 to PND60 [33], with puberty occurring around PND45 [34]. FLX hydrochloride (5 mg/Kg; Fagron, Belgium) was administered via the drinking water for a period of 21 days, followed by a washout period of one week. Control animals received normal tap water. Daily water intake was measured per cage (ml), even as bodyweight of each separate animal (gr). This resulted in an average water intake per gram bodyweight (ml/gr) per day for each animal in a specific cage. Based upon these values and the known fluoxetine concentration in the drinking bottles, the daily fluoxetine consumption per animal was estimated. If needed, the fluoxetine concentration in the drinking water was adjusted to changes in the average water intake (ml/gr) to ensure an intake of FLX of approximately 5 mg/Kg/day [20], [21]. This dosage of 5 mg/Kg is proven to be the minimal dose required for a significant inhibition of 5-HT re-uptake [35]. FLX was given orally in order to mimic the typical route of administration when used clinically. Steady state blood plasma concentrations of FLX and NFLX were assessed for each animal. Blood plasma samples (0.5 ml via the tail vein under short isoflurane anesthesia) were taken in early morning on the 18^th^ day of treatment.

### Stereotactic Surgery

On the day of surgery (3 days post-treatment), the rats were anesthetized with isoflurane (3% induction, 1.5% maintenance within a mixture of oxygen/compressed air (1∶1) with a total flow of 0.3 L/min). The animals were then placed in a Kopf stereotactic apparatus (Kopf Instruments, Tujunga, CA) and kept at a constant temperature using a heating pad. A small incision was made to expose the skull after which lidocaine was used for local anesthesia. A microdialysis guide (MAB 4.15.IC, Microbiotech, Sweden) was placed just above the mPFC (coordinates relative to intra aural point (IAP) and individually corrected for distance IAP-Bregma: ∼12.72 mm; Medial-Lateral: 0.3 mm; Dorsal-ventral from skull: –3.0 mm (adolescent-treated rats) or −4.0 mm (adult-treated rats), according to Paxinos and Watson 1986). A cannula was placed into the right jugular vein, for intravenous administration of the FLX challenge, and tunneled subcutaneous to the skull were it was attached to a stainless steel 20 G curved blunted needle. The jugular vein cannula was filled with a solution of polyvinylpyrrolidone-amoxycycline-heparine (resp.0.9 g/mL, 0.9 mL (48 mg/mL), 0.1 mL (5000 IU/mL)). The probe and jugular vein cannula were fixed to the skull surface with three stainless steel screws and dental glasionomere cement (GC Fuji Plus; GC Europe, Leuven, Belgium). Rats were given subcutaneous Metacam (meloxicam; 1 mg/Kg) post-operatively for pain management and inhibition of inflammation. After surgery, the animals were individually housed.

### In vivo Microdialysis

After a recovery period of 5–6 days, microdialysis experiments were performed during the light cycle (8 or 9 days post-treatment). Experiments were carried out in a separate room with an experimental setup suited for four rats. The setup consisted of four standalone Raturn BASi turntables and corresponding counter-balanced lever arms (Raturn microdialysis setup; BASi, USA), allowing free movement of the animal during microdialysis. Each setup was equipped with several lengths of Peek-tubing (1.2 µL/10 cm), a microdialysis pump (MAB20, Microbiotech, Sweden) and a fraction collector (Univentor, AgnTho’s AB, Sweden). To prevent probe-ultrafiltration, ringer solution was not only pushed but also pulled out of the tubing by the same pump. The microdialysis probes had an exposed membrane of 3 mm (adolescent-treated) or 2 mm (adult-treated animals). Probes were perfused with Ringer solution (147 mM NaCl, 4.0 mM KCl, 1.2 mM CaCl 2 0.7 mM MgCl2,) at a constant flow rate of 1 µL/min beforehand overnight. Early in the morning, rats were transferred to the test room and connected to the setup by tether and peek tubing. Perfusion was kept on for approximately 6 h and samples were collected every 15 min in vials containing 5 µL of antioxidant agent (0.02 N HCOOH (Baker, 6037) en 0.01% cysteine.HCl (Sigma, C-4820)). After an acclimatization period of approximately two hours and one and a half hour of baseline sampling, an acute challenge with FLX (5 mg/Kg in 0.5 ml saline) was given intravenously through the jugular vein cannula. After another one and a half hour of sampling, tetrodotoxin (TTX; 1 µM) was administered via the perfusion medium through the microdialysis probe into the mPFC for the duration of one hour, in order to inhibit neuronal monoamine release. This resulted in six basal level samples, six samples following the acute FLX challenge and four samples following the TTX challenge. All samples were stored at minus 20°C until analysis.

### Analysis of Monoamines

The monoamines 5-HT, DA, and NA and their metabolites (5HIAA, DOPAC, and HVA) were analyzed using a high performance liquid chromatography (HPLC) ALEXYS 100 2D system equipped with electrochemical detection (DECADE II) from ANTEC Leyden (Zoeterwoude, The Netherlands). Samples of 15 µL were spliced in 2 parts of 5 µL and injected on two ALF-115 (150 mm×1 mm, 3 µm C18) columns to analyze dopamine and serotonin and their metabolites. The mobile phase for the ALF-115 columns consisted of 100 mM phosphoric acid, 1 mM KCl, 2.43 mM octanesulphonic acid (OSA) and 16% methanol in milliQ water adjusted to a pH of 3.30. The flow rate was kept constant at 0.035 mL/min. Separation was performed at 38°C and the electrochemical potentials were set at 600 mV against an Ag/AgCl reference in the ISAAC electrochemical cell. The signals were analyzed using Clarity (3.0.3.358) software. The detection limits in a 5 µL sample (signal to noise ratio = 3) were 0.02 nM for the monoamines and DOPAC, 5HIAA and 0.2 nM for HVA.

### Histological Examination

After completion of the experiment, all rats were decapitated after deep CO_2_/O_2_ intoxication. The brains were then removed and snap frozen in isopentane on dry-ice and stored at −20°C until histological examination. Coronal sections of 35 µm were cut on a cryostat system and stained with cresyl violet for verification of the probe placement.

### Plasma Levels of (nor)Fluoxetine

FLX and NFLX were analyzed in serum using LC-MS/MS in the positive ionization mode on a Thermo Scientific (Waltham, USA) Surveyor LC coupled to a Thermo Scientific Quantum Access MS. To 100 µl of serum sample, 250 µl acetonitril/methanol 84∶16 (v/v) containing 400 ng/mL of the internal standard doxepin, was added to precipitate proteins. Samples were vortexed, stored at −20°C for 30 minutes, vortexed again and centrifuged. Of the supernatant, 10 µl was injected onto a Thermo Scientific HyPurity Aquastar (50×2.1 mm, 5 µm) column. A stepwise chromatographic gradient was applied using acetonitril, water and a constant buffer concentration of 0.2% (v/v) ammonium acetate/0.02% (v/v) trifluoracetic acid at pH 3.5. The flow was 0.4 ml/min and the column-oven temperature was 30°C, giving a total chromatographic runtime of 3 minutes per sample, with FLX and NFLX eluting after 2.4 and 2.3 minutes respectively. FLX, NFLX and the internal standard doxepin were measured using the mass transitions 310.1/148.1, 296.1/134.1 and 280.2/107.1 respectively. The method was validated over a range of 20.0–800.0 ng/mL for both compounds. For FLX, the accuracies ranged from 91.5% to 107.8%, the intra-day precisions were below 10.3% and the inter-day precisions were below 17.6%. For NFLX, the accuracies ranged from 93.4% to 109.8%, the intra-day precisions were below 9.2% and the inter-day precisions were below 14.8%.

### Statistical Analysis

Baseline values of extracellular monoamine concentrations were defined as the mean of the five samples before the FLX challenge. All data were then converted to a percentage of that baseline (set as a 100%). All data are presented as mean ± S.E.M. and statistically analyzed using two-way repeated measures analysis of variance (RM-ANOVA). In case of violations of sphericity, Greenhouse-Geisser correction was used. In addition, the AUC-value was determined (area-under-the-curve; sum of percent increases for all post-FLX samples) for each rat. The effects of age at time of treatment and type of treatment was assessed by using age and treatment as the ‘between subjects’ factors and time as the ‘within subjects’ factor. Effects of treatment on bodyweight and water intake were also taken into account. The criterion for significance was set at *p*<0.05. All statistical analyses were performed in SPSS (IBM SPSS Statistics v19.0).

## Results

### Animals

**Figure 1:**
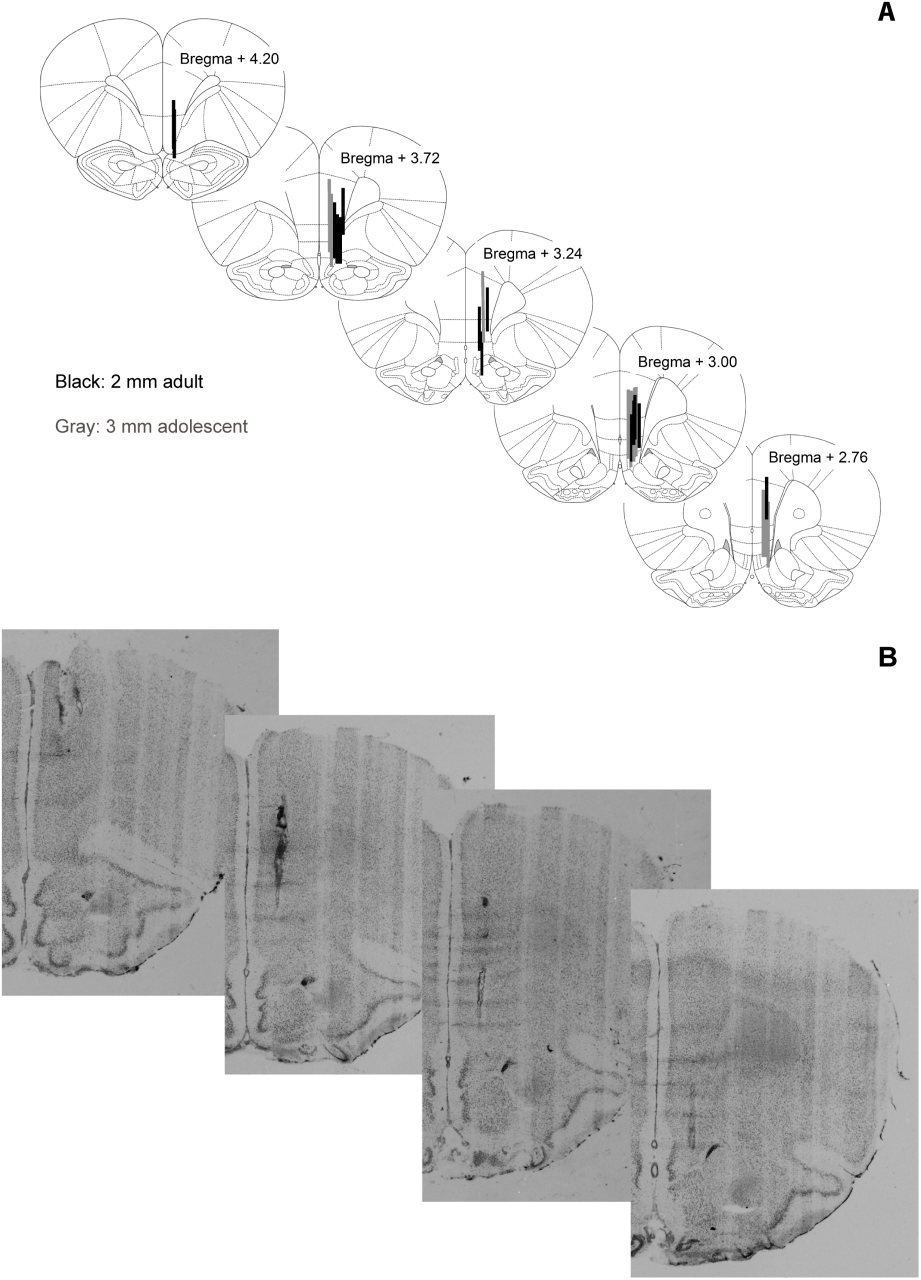
Graphical overview of probe localization. A) Membrane positions of microdialysis probes, based upon histological examination. Length of the membrane was 2 mm (black) in the adults and 3 mm (blue) in the adolescent-treated animals. B) Representative example of probe placement in cresyl blue stained sections.

Correct placement of the microdialysis probe in the mPFC was confirmed for all animals. For a graphical overview of probe placement and a representative histological example we refer to Figure 1. Due to technical issues with the microdialysis setup, two animals of each adolescent-treated group were excluded from further analysis. Additionally, one animal was excluded due to suspected water deprivation caused by blockage of its water bottle on the day before the microdialysis experiment. Animals finally included in the analyses per group: Adolescent FLX-treated (n = 6) and vehicle-treated animals (n = 5), adult FLX-treated (n = 8) and vehicle-treated animals (n = 8).

#### Bodyweight

For both the adult and the adolescent groups, there was no significant difference in bodyweight between the FLX-treated and vehicle-treated animals at the start of treatment (average weight of 312.2 g±8.8 and 70.1 g±4.5, respectively). In the adolescent-treated animals, there was no significant effect of FLX treatment on weight gain. There were no significant differences in bodyweight between the FLX-treated and vehicle-treated animals at the end of treatment, at the time of surgery or at the time of the microdialysis experiments (average weight 252.5 g±17.0). In the adult-treated animals, there was a small but significant effect of treatment on weight gain (RM-ANOVA; time-by-treatment effect (F_23,322_ = 17.46, *p*-value<0.001)). At the end of treatment, FLX-treated animals weighted 378.0 g (±12.8) whereas the adult vehicle-treated animals weighted 391.1 g (±7.2). This difference in bodyweight was no longer present at time of surgery or at time of the microdialysis experiments (average weight 403.1 g±11.0).

#### Water intake

For both the adult and the adolescent groups, there was no difference in water intake between the FLX-treated and vehicle-treated animals during the acclimatization period, thus before start of treatment. Relative water intake was significantly higher in younger animals than in the adult animals (average water intake of 0.127 mL/g±0.01 and 0.073 mL/g±0.01, respectively) and water intake also significantly decreased with aging in the young animals. There was a clear effect of FLX on water intake, especially in the adult animals. In young animals, water intake was on average 8.7% lower in the FLX-treated animals (n.s.). In the adult groups, this difference was 30.5% (*p*-value<0.001). This is most probably due to the taste of the drinking water being affected by FLX and the concentration of FLX in the drinking water being higher in the adults. Since no major weight loss (over 10% weight loss or less weight gained compared to controls) was observed at any time point, we believe that water intake was sufficient for all animals.

#### Drug treatment

On average, the adult animals received a dosage of 131.1 mg/l FLX hydrochloride per day. With an average daily water intake of 0.041 mL/g, this resulted in an average consumption of 5.31 mg/kg (±0.21) FLX hydrochloride per day. The adolescent animals received an average dosage of 52.8 mg/l hydrofluoxetine. With a daily average water intake of 0.097 mL/g, this resulted in an average consumption of 5.03 mg/Kg (±0.42) FLX hydrochloride per day for the adolescent-treated animals. There was no significant difference in drug intake between the two age groups (*p*-value>0.10). Total plasma concentration of active components (FLX plus NFLX) on day 18 of treatment was (on average) 262.13 ng/mL (±49.0) for the adult-treated animals and 124.67 ng/mL (±9.6) for the adolescent-treated animals. Plasma concentrations of FLX alone were below the official detection limit of 20 ng/mL for all adolescent-treated animals and just above or around 20 ng/mL for most of the adult-treated animals.

### Microdialysis

#### Serotonin

**Figure 2:**
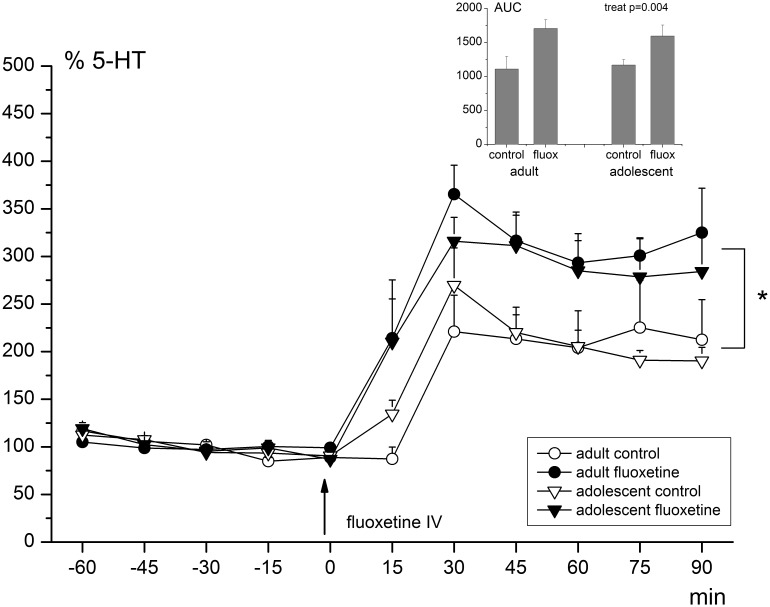
Extracellular 5-HT levels in the mPFC before and after acute FLX challenge. Levels are given as percentage (%) of the average baseline value ( = 100%). 5-HT release was significantly increased after the acute FLX challenge in all groups (RM-ANOVA; effect of time (F_10,230_ = 48.342, *p*-value<0.001) and this increase was significantly enhanced in the chronically treated animals (RM-ANOVA; effect of time-by-treatment; F_10,230_ = 4.417, *p*-value = 0.007) and reaches its maximum after approximately 30 minutes. The inset shows the AUC-values per group (effect of treatment; F_1,27_ = 10.333, *p*-value = 0.004). Black arrow indicates the time point of the acute FLX challenge (5 mg/kg, i.v.). Error bars represent one S.E.M.

Basal levels of 5-HT did not differ between groups; there were no statistically significant effects of age, treatment or age-by-treatment (Table 1; *p-*values>0.100). On average, the basal level of extracellular 5-HT in the mPFC was 0.170 nM (±0.067). The acute i.v. FLX challenge had a significantly increasing effect on 5-HT release compared to baseline values in all groups (RM-ANOVA; effect of time (F_10,230_ = 48.342, *p*-value<0.001); see also Figure 2). Additionally, there was a significant effect of treatment within the repeated-measures analysis (effect of time-by-treatment; F_10,230_ = 4.417, *p*-value = 0.007) as well as within the AUC-values (effect of treatment; F_1,27_ = 10.333, *p*-value = 0.004). The increase in 5-HT concentrations compared to baseline was on average 197% in the vehicle-treated animals, while it was 293% in the FLX-treated animals. There were no age or age-by-treatment effects, neither in the repeated measures (age: F_1,23_ = 0.055; *p*-value = 0.817; age x treatment: F_1,23_ = 0.255; *p*-value = 0.618), nor in the AUC-data (age: F_1,27_ = 0.026; *p*-value = 0.874; age x treatment: F_1,27_ = 0.279; *p*-value = 0.602). Since the levels of 5HIAA were out of range for several animals, changes in 5HIAA concentrations could not be determined. Therefore, 5-HIAA levels were not analyzed.

**Table 1:** Basal monoamine levels in the mPFC per group.

Monoamine/	Adult control	Adult FLX	Adolescent control	Adolescent FLX	Total
Metabolite	nM ± SD (n)	nM ± SD (n)	nM ± SD (n)	nM ± SD (n)	nM ± SD (N)
**5-HT**	0.180 nM ±0.081 (8)	0.158 nM ±0.048 (8)	0.166 nM ±0.064 (5)	0.176 nM ±0.086 (6)	0.170 nM±0.067 (27)#
**DA**	0.100 nM ±0.068 (8)	0.182 nM ±0.115 (8)	0.200 nM ±0.120 (5)	0.196 nM ±0.127 (6)	0.168 nM±0.110 (27)#
**DOPAC**	4.649 nM ±5.150 (8)	8.610 nM ±5.620 (8)	4.524 nM ±3.187 (5)	8.256 nM ±7.895 (6)	6.601 nM ±5.751 (27)#
**HVA**	6.453 nM ±5.106 (8)	9.610 nM ±4.527 (8)	6.046 nM ±3.080 (5)	9.095 nM ±5.480 (6)	7.900 nM ±4,722 (27)#
**NA**	0.179 nM ±0.113 (8)	0.296 nM ±0.186 (8)	0.152 nM ±0.097 (2)	0.217 nM ±0.185 (3)	0.226 nM ±0.154 (21)$

# No significant effect of age, treatment or age-by-treatment between groups;

$ Group analysis not performed due to low amount of subjects in the adolescent-treated groups.

For each monoamine or metabolite, the average baseline concentrations (nM ± SD (n)) are given per group and for all animals together.

#### Dopamine

**Figure 3:**
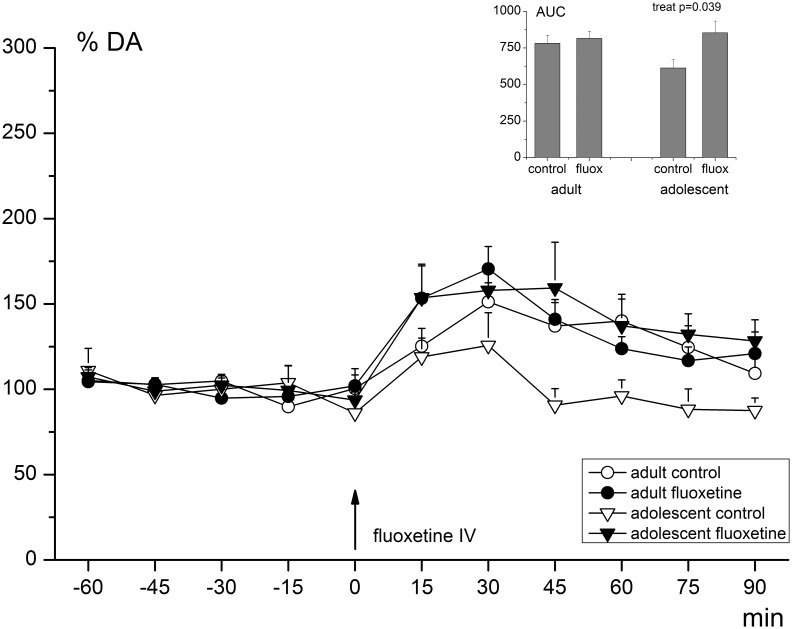
Extracellular DA levels in the mPFC before and after acute FLX challenge. Levels are given as percentage (%) of the average baseline value ( = 100%). DA release was significantly increased after the acute FLX challenge in all groups (RM-ANOVA; effect of time (F_10,190_ = 12.037, *p*-value<0.001) and reaches its maximum after approximately 30 minutes. The AUC values (inset) showed a significant effect of treatment (increased release in treated animals), this was however not confirmed by the RM-ANOVA analysis (no time-by-treatment effect; *p*-value>0.100). Black arrow indicates the time point of the acute FLX challenge (5 mg/kg, i.v.). Error bars represent one S.E.M.

In four adult-treated animals, DA values were not quantifiable. Therefore, both the adult FLX-treated and the adult vehicle-treated group consisted of six animals in the DA analysis. Basal levels of DA did not differ between groups; there were no statistically significant effects of age, treatment or age-by-treatment (Table 1; *p-*values>0.100). On average, the basal level of extracellular DA in the mPFC was 0.168 nM (±0.110). The acute FLX challenge had a significantly increasing effect on DA release compared to baseline values in all groups (RM-ANOVA; effect of time (F_10,190_ = 12.037, *p*-value<0.001); see also Figure 3). Similar as with the 5-HT, the increase in DA concentrations compared to baseline values was on average lower in the vehicle-treated animals than in the FLX-treated animals (118% vs. 141%). This difference reached statistical significance within the AUC-analysis (treatment effect; F_1,23_ = 4.914, *p*-value = 0.039), but not within the repeated-measures analysis (effect of time-by-treatment (F_10,190_ = 1.878, *p*-value = 0.113); see Figure 3). There were no age or age-by-treatment effects, neither in the repeated measures (age: F_1,19_ = 1.117; *p*-value = 0.304; age x treatment: F_1,19_ = 2.912; *p*-value = 0.104), nor in the AUC-data (age: F_1,23_ = 1.113; *p*-value = 0.305; age×treatment: F_1,23_ = 2.822; *p*-value = 0.109). Although the FLX treated animals tended to have both higher baseline DOPAC and HVA values, there were no significant effects of either treatment or age for both DA metabolites (Table 1; *p*-values>0.100). The average basal amount of DOPAC was 6,601 nM (+/−5,751) and 7,900 nM (+/−4,722) for HVA. There was no significant effect of the acute challenge on either DOPAC or HVA values in any of the groups. Average DOPAC and HVA levels after the challenge were 6,641 nM (+/−5,988) and 6,903 nM (+/−4,629), respectively.

#### Noradrenaline

**Figure 4:**
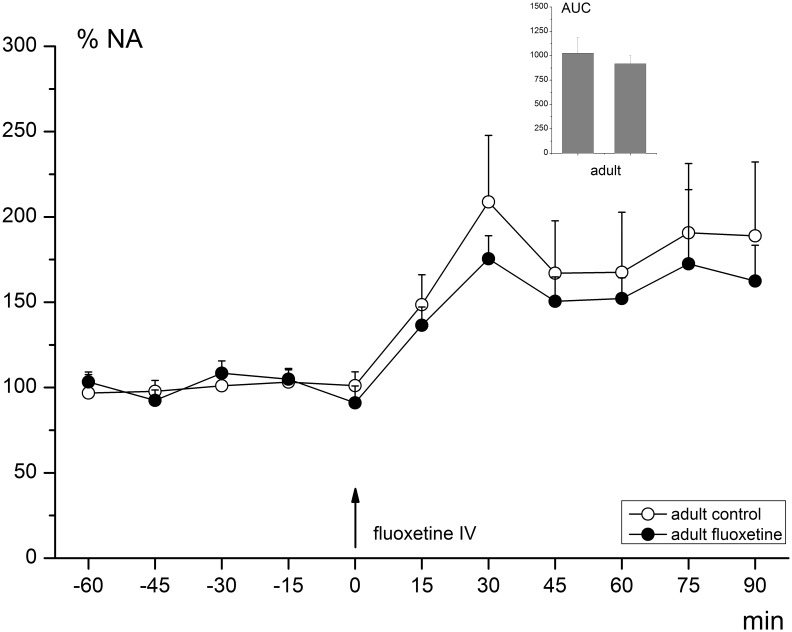
Extracellular NA levels in the mPFC before and after acute FLX challenge. Levels are given as percentage (%) of the average baseline value ( = 100%). NA release after the challenge is significantly enhanced (RM-ANOVA; effect of time (F_10,200_ = 12.197, *p*-value<0.001) and reaches its maximum after approximately 30 minutes.

Due to chromatographic problems in the analysis of NA for several of the adolescent-treated animals, NA values were not compared on group level. However, again there was a clear overall effect of the FLX challenge on NA release compared to baseline values (RM-ANOVA; effect of time (F_10,200_ = 12.197, *p*-value<0.001); average increase of 170%). The basal level of extracellular NA was on average 0.226 nM (±0.154) (See Table 1 for basal NA concentrations per group). After the challenge, the average NA level was nM 0.360 (±0.240). The effect of the acute challenge on adult NA levels can be found in Figure 4.

#### Tetrodotoxin

After one hour, TTX (1 µM in the perfusion fluid) had reduced extracellular 5-HT in the frontal cortex to 91% of the baseline values (after an initial increase of on average 247% caused by the FLX challenge). Extracellular levels of DA and NA were reduced even further to respectively 38% and 17% of the baseline values, showing that the monoamine release was indeed inhibited by TTX.

## Discussion

We have examined the long-term effects of chronic FLX treatment on extracellular monoamine concentrations in the mPFC of adult- and adolescent-treated rats. After a washout period of one week, basal extracellular levels of 5-HT, DA and NA did not differ between groups. Acute FLX caused an increase in both 5-HT and DA and NA release in all animals. The effects of prior FLX treatment on the 5-HT response to an acute challenge were clearly visible in both age groups, in that FLX-treated rats showed a twofold higher increase than saline-treated animals. A similar effect was seen on the DA response. Age-at-treatment did not influence these results.

### Enhancing Effect of Chronic Fluoxetine on 5-HT Release after One-week Washout

The increase in extracellular 5-HT levels produced by acute SERT blockade was significantly enhanced one week following chronic treatment. This study is the first microdialysis study looking at FLX effects to use such a long washout period. Previous studies have investigated the effects of chronic FLX treatment on 5-HT system responsiveness with shorter wash-out periods, ranging from 1–3 days. Rutter et al. (1994) and Invernizzi et al. (1996) showed that chronically treated animals have higher basal 5-HT levels 24 h after the last dosage while the latter authors reported that this effect is absent 96 h post treatment. The increases in basal levels within 24 hours after the last dosage are most probably due to reuptake blockade by the residuals of active NFLX in the brain, which can remain present up until 48 h after the last administration [36]. Here we showed that after a washout period of one week the initial increase in basal 5-HT concentrations is gone, but that a hypersensitive response to 5-HT uptake inhibition remains. In previous studies, a challenge given 24 h after the final dose hardly further increased the still high levels in chronically FLX-treated rats [24], [31] but two days later, the effect of the challenge was the same in FLX- and saline-pretreated animals [31]. Several factors could explain the difference with our results – the treatment protocol (10 mg/kg i.p. once daily for 14 days in the Invernizzi study versus 5 mg/kg via drinking water for 21 days in our case), the acute challenge (Invernizzi: 10 mg/kg i.p. versus 5 mg/kg i.v.) and the position of the microdialysis probe (Invernizzi: lateral frontal cortex vs mPFC; cf [37]) may have resulted in a more constant FLX exposure and in more sensitive conditions to detect FLX-induced effects. As said before, extracellular 5-HT release is regulated by presynaptic 5-HT receptors in the raphe nuclei. Several studies have shown desensitization (loss of function) of the 5-HT1A and 5-HT1B autoreceptor after chronic SSRI treatment [29], [38]. Although this does not seem to affect basal 5-HT concentrations, it may facilitate postsynaptic processes in stimulation of the serotonergic system. In addition, chronic treatment may have upregulated postsynaptic receptor expression. Both processes could account for the enhanced 5-HT release after an acute FLX challenge seen in chronically treated animals. Since resensitization of internalized receptors is suggested by several studies to require the synthesis of completely new receptors, or the replenishment of regulatory proteins, these processes can take much longer than one week after treatment discontinuation to normalize [29], [31], [39].

### No Apparent Age-at-treatment Effects

We expected to find different effects of chronic treatment in adolescent-treated animals compared to adult-treated animals. However, such differences were not observed. As previously mentioned, a recent phMRI study by our group [18] suggested age-related differences in 5-HT system responsiveness to an acute SSRI challenge after a similar treatment regime and washout period as used in the current study. Apparently, these effects are not caused by differences in extracellular 5-HT concentrations, since in the present study we demonstrated that the same extracellular concentrations were present in *all* treated animals, irrespective of age-at-treatment, both before and after the acute challenge. In conclusion, there were no apparent age-by-treatment effects of chronic FLX on basal extracellular 5-HT concentration and on the FLX-induced increase. Still, we cannot rule out that there are age-related differences in more down-stream processes such as post-synaptic receptor sensitivity [26]. In this way, similar extracellular 5-HT concentrations may have dissimilar effects on 5-HT transmission, which might still explain our earlier phMRI findings where adolescent-treated showed an increased response to an acute challenge, while adult-treated animals were less responsive [18]. Also, since resensitization of the autoreceptors presumably takes longer than the one-week washout applied here, it might be that there are age-related differences or even non-transient changes within this resensitization process. To rule this out, even longer washout periods might be needed.

### Effects on Other Monoamines

As hypothesized, a clear effect of the acute FLX challenge was observed on the release of extracellular DA and NA, while no effect of chronic FLX treatment on their basal levels was seen. Unpredicted however, was the small but statistically significant effect of the preceding chronic FLX treatment on DA release after the acute challenge in both age groups, just as was seen with 5-HT. Previous studies have shown that FLX can indeed acutely affect DA and NA release in the prefrontal cortex, presumably via the 5-HT2c receptor and/or post-synaptic 5-HT1A receptor [30], [40], but other studies have reported no effects on DA and NA concentrations after an acute challenge [41] or directly after chronic administration [42], [43]. Although this effect of previous FLX treatment on the DA response to an acute 5-HT challenge is thus less easily explained, it is clear that almost all 5-HT receptors are capable of modulating DA release [44] and it is therefore not surprising that, considering the effects seen in the 5-HT system, also the DA system is similarly affected. It was also previously shown that chronic FLX treatment requires long-term recovery of DA metabolism, just as is the case with 5-HT [45]. Our findings thus further support the possible long-lasting interference of FLX on DA activity. Similar to our results, it was previously shown that there is little to no effect of an acute FLX challenge on extracellular DOPAC and HVA in the PFC [46], [47], although the study by Li et al. (1999) also fails to report changes in DA release. We may even need to look at yet other neurotransmitters systems than 5-HT, DA and NA. For instance, GABA and glutamate projections are also known to be involved in 5-HT feedback control [26]. Studies on the effect of chronic FLX treatment on these monoamines and their metabolites are however even more scarce.

### Strengths and Limitations

So far, there is only a small number of studies that has looked into the ongoing effects of chronic FLX treatment on neurotransmitter responsiveness after its cessation. Our results show that the 5-HT system remains sensitive to external changes quite long after complete washout of the drug. While it is known from stimulant drugs that repeated administration may lead to long-lasting sensitization of the DA system [48], similar studies in the 5-HT system were absent. Even though less invasive effects are to be expected from uptake inhibitors than from stimulants such as amphetamine that both inhibit uptake and actively force neurotransmitter release, it would still be worthwhile to further investigate this in future research. This type of studies will give us more insight in the 5-HT system and on the understanding of the effects of SSRIs thereupon. On a more future prospect, these studies might also have implications for clinical practice. For instance, although speculative, the switch between SSRIs in case of treatment non-response, as is currently described in clinical guidelines, may not be due to superior effect of the alternating SSRI but to the hypersensitivity of the 5-HT system induced by the previous one. Such a mechanism might be explained by our current results. Evidently, further research is needed to confirm and support this reflection.

A possible limitation of the present study is that we only studied the effects in one brain area, the mPFC. We should be aware that region-specific differences in 5-HT function and in response to SSRIs exist and that our findings might not be directly translatable to other brain regions, such as the raphe nuclei or the dorsal hippocampus, which is one of the other major terminal regions of 5-HT projections from the raphe [47], [49]. Also, only male rats were included in this study, while it is known that in humans, (adult) depression and thus SSRI treatment is more common in women than in men. If this study is to have implications for clinical practice, it would be of interest to see if similar findings are obtained in female subjects. However, since our primary goal was to investigate the effects of age on possible long-term effect of fluoxetine on monoamine levels and because the hormonal cycle of female subjects, and especially the differences herein between young and adult females, would have introduced additional variation to our results, as sex hormones are known to influence monoamine function [50], only male subjects were chosen for this study. Another limitation might be that it turned out to be very difficult to come to similar conditions of chronic treatment in adolescent and adult rats. The FLX treatment led to reduced water intake (presumably due to the taste of the drug) and also to a small reduction in weight gain in the adult-treated rats only. Although FLX is known for its weight reducing capacities [51], this small reduction in weight gain could also be due to the decreased water intake, since reductions in water intake will subsequently lead to reduced food intake [52], [53]. Still, water and food intake was considered sufficient for all animals and no actual weight loss was observed. Also, the adult-treated animals ingested similar FLX concentrations as the adolescent animals. Yet, the combined plasma concentrations of FLX and NFLX of the adult-treated animals were twofold of those seen in the adolescent-treated rats. This is probably due to faster drug metabolization in younger individuals [54]. Although the same dosage and route of administration induced similar plasma levels of FLX and its active metabolite NFLX in both adolescent and adult rats in a previous study [20], similar age-related differences in drug plasma levels have been described with the oral intake of paroxetine [21]. A consequence of these differences in metabolization rate could be that the (N)FLX plasma levels of the younger animals varied more over the course of one day than was the case in the adult animals. Although not likely, we cannot rule out that this has affected the outcome of our study. Also, while plasma (N)FLX levels in the adult rats were within the therapeutic range for humans (160–560 ng/mL; [55]), levels of the adolescent animals were on the low side, making it arguable whether the (N)FLX concentrations in adolescents (114–139 ng/mL) were sufficient in terms of therapeutic efficacy. Still, the aforementioned therapeutic range is based upon adult values and thus not readily translatable to children and adolescents [54]. Additionally, it remains difficult to directly convert human therapeutic values to their efficacy in rats, since FLX metabolizes much faster in rats than in humans [36]. Unfortunately, very little is known on the pharmacokinetics of FLX in both children and young rodents.

### Conclusions

Our findings indicate ongoing effects of FLX treatment on 5-HT regulation one week after drug discontinuation. After the washout period, basal extracellular 5-HT levels are normalized, indicating that the direct effects of chronic FLX on 5-HT concentrations in the rat mPFC are gone. However, when challenging the 5-HT system, we observed a hyper-responsive 5-HT system in adult- as well as adolescent-treated animals and to a lesser extent the same effect was seen on the DA system. It thus seems that both pre- and postsynaptic feedback mechanisms that should maintain stable monoamine levels are not yet completely restored one week after treatment discontinuation. These findings are of relevance, since they give us more insight in the working mechanisms of the 5-HT system and enhance our understanding of the effects of SSRIs thereupon, and might, on the long-term, have implications for clinical practice. Moreover, we can conclude that age-related difference in extracellular concentrations of 5-HT or in 5-HT responsiveness are not likely an explanation for the observations we made earlier with phMRI. Future studies focusing on more down-stream (post-synaptic) processes of 5-HT transmission, or even on other neurotransmitter systems, or studies using an even longer washout period to ensure complete restoration of the feedback mechanisms should establish if there are age-dependent changes in these specific processes.
